# Modeling dose uncertainty in cone-beam computed tomography: Predictive approach for deep learning-based synthetic computed tomography generation

**DOI:** 10.1016/j.phro.2025.100704

**Published:** 2025-01-26

**Authors:** Cédric Hémon, Lucía Cubero, Valentin Boussot, Romane-Alize Martin, Blanche Texier, Joël Castelli, Renaud de Crevoisier, Anaïs Barateau, Caroline Lafond, Jean-Claude Nunes

**Affiliations:** Univ. Rennes, CLCC Eugène Marquis, INSERM, LTSI - UMR 1099, F-35000 Rennes, France

**Keywords:** CBCT-to-CT generation, Uncertainty estimation, Dose uncertainty, Head and Neck

## Abstract

**Background and purpose:**

Cone-beam computed tomography (CBCT) is essential in image-guided radiotherapy (RT) for patient positioning and daily dose calculation. However, CT numbers in CBCT fluctuate and differ from those in computed tomography (CT), requiring synthetic CT (sCT) generation to improve dose calculation accuracy. CBCT-to-sCT synthesis remains a challenging and uncertain task in clinical practice. This study aims to introduce a voxel-wise uncertainty estimator correlated with the error between sCT and CT.

**Material and Methods:**

Eighty-five head and neck (H&N) patients treated with photon RT from a single center were selected for developing and validating our uncertainty estimation method. To test the method’s robustness on out-of-distribution images, three additional patients from different centers were included. Our proposed uncertainty estimation method builds on established conventional techniques. Additionally, to explore potential error scenarios, we generated several ‘plausible’ sCTs representing variations in sCT generation caused by CBCT quality differences. This allowed us to quantify dose uncertainties.

**Results:**

The effectiveness of uncertainty maps was evaluated by correlating them with the absolute error map between sCT and CT, yielding a Pearson correlation coefficient between 0.65 and 0.72. Dose uncertainty was determined on the dose-volume histogram (DVH). For all patients except one, the reference CT DVH was included in the uncertainty interval defined by the sCT-derived DVH.

**Conclusions:**

Our proposed methods effectively predict uncertainty maps that aid in evaluating sCT quality. This approach also provides a novel method for estimating dose uncertainty by defining a confidence interval around the CT DVH using the estimated sCT uncertainty.

## 1 Introduction

In clinical practice, cone-beam computed tomography (CBCT) is essential for volumetric data acquisition in image-guided radiotherapy (IGRT). However, it suffers from limited field of view (FOV) and fluctuating computed tomography (CT) numbers compared to standard CT [1]. Indeed, CBCT images are subject to several artifacts [2].

These limitations hinder tasks like segmentation and dose calculation in adaptive radiotherapy (ART). Although there are several techniques for improving CBCT image quality [3], [4], [5], analytical and Monte-Carlo (MC) methods face time constraints, prompting the use of machine learning for image quality improvement and reconstruction [3].

Among these techniques, deep learning (DL) models have shown promise in the generation of synthetic CT (sCT) with high accuracy and efficiency [4], [6], [7]. In the realm of DL-based image translation, the most widely used techniques include GANs, CycleGANs, Transformers, and, more recently, diffusion models [6], [7]. Other specialized approaches, such as using GANs for low-dose CT denoising and MRI super-resolution enhancement, are also being explored [6].

Synthetic generation in medical imaging remains affected by uncertainties [8]. As recommended by Claessens et al. [9], an essential aspect for clinical applications is incorporating an additional output channel for predicting uncertainty maps [10]. This output channel helps identify CBCT images that deviate from expected distributions. Moreover, it helps in predicting potential errors in the resulting output image (sCT).

Uncertainty can be categorized into two types [8]: data-dependent (aleatoric), which arises from noisy inputs or imprecise labels, and model-dependent (epistemic), which is due to variability in model parameters.

Bayesian methods are mathematically suitable for uncertainty estimation but are computationally expensive and challenging to optimize effectively in practice. Several methods have been proposed for uncertainty estimation, such as ensemble learning [11], [12], test-time augmentation (TTA) [13], Bayesian approximation methods like MC dropout (MCD) [10], [13], [14], [15], [16], and heteroscedastic loss [14], [16], [17]. Ensemble learning offers high performance but is resource-intensive [18]. TTA provides a practical means for improving aleatoric uncertainty estimation by generating multiple augmented versions of input data [19]. Meanwhile, MCD facilitates epistemic uncertainty estimation by leveraging dropout layers during inference [10], [14]. TTA and MCD can be applied to pre-trained models. Heteroscedastic loss can improve aleatoric uncertainty handling, though it is sensitive to model architecture [20].

Building on previous work [21], [22] in uncertainty estimation, we introduce a novel contribution in radiotherapy: voxel-level dose uncertainty estimation. Unlike existing studies [10], [11], [12], [13], [14], [15], [16] that focus primarily on voxel-level sCT uncertainty, our method directly links sCT uncertainty to dose uncertainty. By combining MCD and heteroscedastic loss, both aleatoric and epistemic uncertainties without separate sampling were predicted. Furthermore, dose uncertainties were quantified by generating several ‘plausible’ sCTs through MC sampling of estimated uncertainties. An innovation of our work is the assessment of the generalizability of these uncertainty estimates in out-of-distribution (OOD) scenarios, achieved by using patient data from various centers with different challenges like noise levels, FOV constraints, and anatomical artifacts.

## 2 Material and methods

**Fig. 1:**
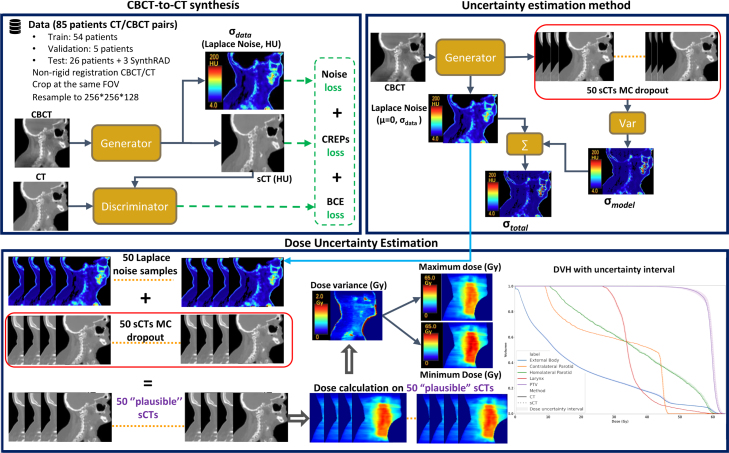
Workflow for predicting sCT and its uncertainty regarding CT numbers and dose prediction. First, images were preprocessed, then divided into a cohort to train the model. In the training phase, the generator (described in 2.3) predicts both the sCT and its uncertainty map ($\sigma_{data}$) by minimizing the noise loss (Eq. (1)). The uncertainty of the model ($\sigma_{model}$) is estimated by generating 50 sCTs using Monte-Carlo dropout injection during inference. For the Uncertainty estimation method (upper right corner), the color bars represent uncertainty values in HU. For dose uncertainty estimation, these 50 sCTs are perturbed by Laplacian random noise based on the uncertainty ($\sigma_{data}$) estimated by the generator. These perturbed sCTs (50 “plausible” sCT) are then used to generate a corresponding set of 50 distinct dose distributions. By analyzing the variance among these doses, three distinct dose values were computed: the average dose, the minimum dose, and the maximum dose. The minimum and maximum doses were defined as $\left[{\mathrm{Dose}}_{\mathrm{sCT}}-3\sigma_{dose},{\mathrm{Dose}}_{\mathrm{sCT}}+3\sigma_{dose}\right]$, where $\sigma_{dose}$ is the dose standard deviation. This approach enables the creation of a DVH that incorporates the dose uncertainty interval.

Fig. 1 presents the workflow of this study on CBCT-to-CT synthesis with uncertainty prediction.

### 2.1 Dataset

**Fig. 2:**
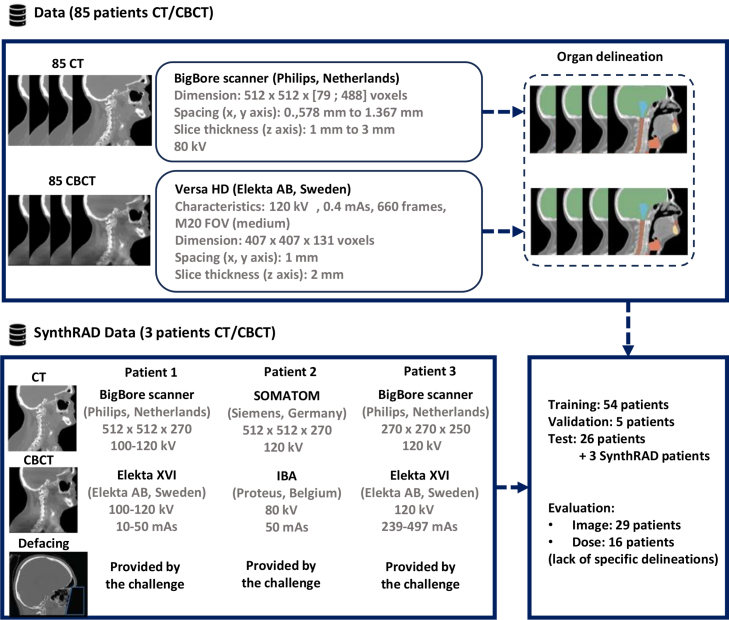
Overview of the dataset used in this study. This dataset includes 85 patients, each with CT and CBCT scans, delineated according to international guidelines [24]. These patients were divided into training (54), validation (5), and test (26) sets. The test set also included three additional SynthRAD patients [23] to test the robustness of the method on OOD data. A defacing of SynthRAD CBCT/CT data was performed to anonymize the patient. The defacing was performed using the contours of the eyes by removing voxels inferior and anterior to the eyes. For the dose calculation analysis, only 16 of the 26 patients originally tested were used. This decision was made due to the time-intensive nature of the dose calculation process, combined with the robustness and consistently low variability observed in dose-level results across different patients.

For this study, 85 patients with locally advanced oropharyngeal carcinomas treated with external beam RT were retrospectively selected. A personalized thermoplastic head and shoulder mask with five fixation points ensured proper patient positioning, with head support provided by a cushion. Weekly CBCTs were acquired, but only the CBCT acquired with a time interval close to the CT was selected. These patients were divided into training (54), validation (5), and test (26) sets. The test set also included three additional patients from the SynthRAD database to test the robustness of the method on OOD data [23]. Details on the dataset and patient characteristics are provided in Fig. 2.

### 2.2 Image preprocessing

To ensure correct alignment between CT and CBCT, the CBCT was registered to the CT using bone-based rigid registration, followed by non-rigid registration with VoxelMorph (Hybrid) [25], [26]. CBCT/CT deformable registration adjusts CBCT geometry to align with CT for accurate sCT generation. CBCT contours were generated by applying the inverse deformable vector field, obtained from the deformable registration, to the manually segmented CT. To maintain anatomical accuracy, the CT and CBCT images were acquired within a short interval of each other (typically the same day) and with regularization of the deformation field to minimize excessive deformation.

For each patient, CT and CBCT images were cropped to the smallest CBCT FOV (260 × 197 × 123) and resized to 256 × 256 × 128 voxels using a third-order B-Spline interpolation. The resampled CT and CBCT had resolutions ranging from $1.38\times 0.86\times 1.77mm^{3}$ to $1.61\times 1.14\times 1.95mm^{3}$. Before training, CT numbers were clipped to the range [−1000, 3000] HU and normalized by a factor of 1000.

### 2.3 CBCT-to-CT synthesis

The proposed algorithm to generate both sCT and uncertainty maps from CBCT was based on a 3D cGAN, initially introduced in the SynthRAD2023 challenge (BreizhCT team) [21], [22]. To enable uncertainty estimation, the original method was modified by adding a second output channel with a convolutional layer designed to predict the standard deviation of input’s noise, representing the uncertainty map ($\sigma_{data}$) associated with it. Furthermore, the training strategy was adjusted to use supervised learning instead of an unsupervised approach [21], [22], and the entire image was used for training instead of a patch-based approach. All other aspects of the original method remain unchanged, and further details can be found in Supplementary CBCT-to-CT synthesis subsection.

### 2.4 Uncertainty estimation method

Ideally, uncertainty estimation revolves around the posterior probability distribution p(Y|X), specifically focusing on its variance. The variable Y represents the output predictions, while X denotes input samples. In neural networks, due to the large number of (possibly non-linear) operations required to generate predictions, the exact posterior distribution is intractable.

This uncertainty assessment method combines two noise variance estimators: one for the input and one for the weights W. The total variance, $\sigma_{tot}=\sqrt{\sigma_{data}^{2}+\sigma_{model}^{2}}$, combines aleatoric and epistemic uncertainties, data and model noise are described in the following paragraph. The variance of each noise component is quadratically summed to calculate the total noise variance. Quadratic summation of uncertainties reduces the risk of overestimating total error by considering that simultaneous extreme error values are rare. Quantifying data uncertainties requires assuming a specific noise distribution, here modeled by a Laplacian distribution. The network is trained to minimize the following loss function, denoted as $L_{unc}$, which computes the logarithm of the standard deviation $\sigma_{data}\left(x\right)$:

(1) $$L_{unc}=\frac{1}{N}\overset{N}{\underset{i=0}{\sum }}\frac{\left|CT_{i}-G\left(CBCT_{i}\right)\right|}{\sigma_{data_{i}}\left(CBCT_{i}\right)}+\log\left(\sigma_{data_{i}}\left(CBCT_{i}\right)\right)$$

$\sigma_{data_{i}}\left(x\right)$ represents the data-based uncertainty for a specific voxel i, predicted by the network during training. The summation accounts for all voxels, with N representing the total number of voxels in the dataset. During training, the presence of $L_{unc}$ loss influences sCT synthesis. Although the supervised training is sensitive to registration errors, adopting the Laplacian distribution offers greater robustness to outliers, along with a sparsity property [27]. The Laplacian distribution assigns less weight to extreme values than other distributions, thanks to its heavier tails, making the model more resilient to outliers. Moreover, it encourages sparsity by encouraging solutions where many parameters are zero or near zero, a characteristic attributed to the distribution’s sharp peak at the mean.

Similarly to aleatoric approaches, epistemic uncertainty is represented by introducing a distribution over the weights W, within the neural network. The weight distribution after training is represented as p(W|X,Y). However, obtaining the exact posterior distribution is computationally infeasible, so we approximate it using MCD. This involves applying dropout injection during inference, without using it during training to effectively sample from the weight distribution. In our case, we performed 50 forward passes with a dropout rate of 0.3.

Formally, the posterior distribution p(W|X,Y) is approximated by q(W;ϕ), where ϕ refers to the dropout rates applied to the weights. This approximation is interpreted as a Bernoulli distribution over the weights and allows us to estimate the uncertainty in the model’s predictions by computing the variance of the outputs across the sampled weight configurations.

The model uncertainty for a single prediction is represented by the variance $\sigma_{model}^{2}$, calculated as:

(2) $$Var_{model}\left(y\right)=\sigma_{model}^{2}=\frac{1}{T}\overset{T}{\underset{t=1}{\sum }}{\left(y_{t}-\bar{y}\right)}^{2}$$

Here, $Y_{t}$ is a set of T sampled outputs for weight instances q(W;ϕ). $\bar{y}$ denotes the empirical mean of the generated sCTs.

After estimating the uncertainty, a calibration process was applied to adjust the dropout-based uncertainties, which initially lacked proper calibration. This process involved aligning the estimated uncertainties with the actual errors observed in the training dataset using linear regression. During inference, the regression model is used to adjust the predicted uncertainties, based on the calibration performed during the training phase.

### 2.5 Dose uncertainty estimation using ‘plausible’ sCTs

To assess the impact of generation uncertainties on dose calculation, a MC approximation strategy was used to estimate dose variance by generating multiple ‘plausible’ sCTs (p-sCTs). These p-sCTs were created to represent potential error scenarios, capturing variations in sCT quality. Specifically, p-sCTs were generated using the two variance estimators: sCTs generated by MCD were directly used in the estimation, and additional p-sCTs were created by perturbing these sCTs with random noise based on the Laplacian noise estimated by the network (Section 2.7).

### 2.6 Network implementation

The DL methods were implemented in Python 3.8 using PyTorch 1.12 with CUDA 11.7. The Adam optimizer was used with a batch size of 1 and a learning rate of 0.0002. The model was trained for 200 epochs. The estimation of $\sigma_{tot}$ took 14 s during inference, using 50 samples for MCD.

### 2.7 Evaluation protocol

**Fig. 3:**
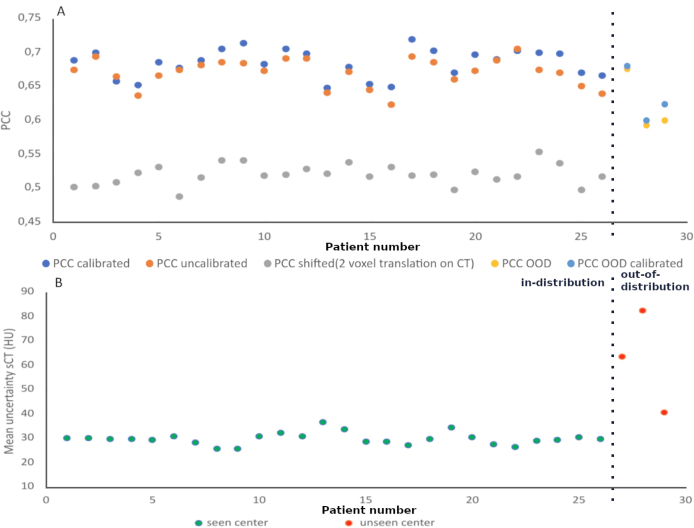
Uncertainty results are detailed depending on the scenario described. In-distribution and OOD datasets were used. **Subplot A:** The Pearson correlation coefficient between the uncertainty map and the absolute error map (|CT−sCT|) was computed for test patients in three scenarios: (1) before and after model calibration, (2) after applying a 2-voxel translation to the CT on each axis, and (3) patients from an unseen center before and after model calibration (different from the training center). **Subplot B:** The mean uncertainty of the sCT was presented according to test patients, categorized by whether the center was seen (green) or unseen (red). The mean uncertainty is calculated as the average uncertainty across all voxels within the external body region. (For interpretation of the references to color in this figure legend, the reader is referred to the web version of this article.)

**Fig. 4:**
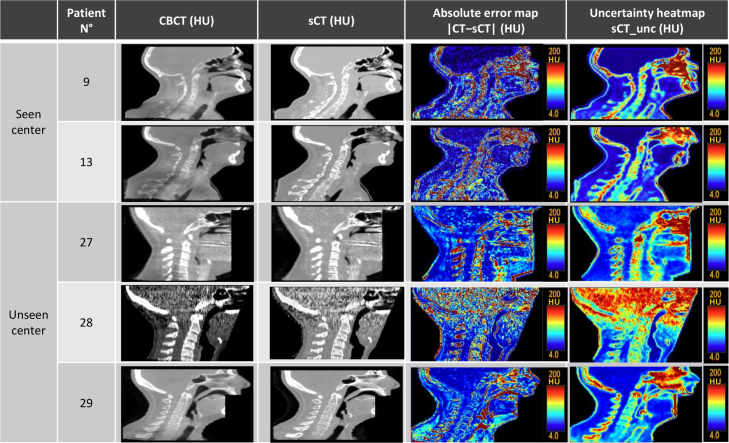
Results of total uncertainty maps for five test patients. The Columns are : CBCT, sCT, absolute error map between sCT and CT, and sCT total uncertainty heatmap. Patients 27, 28, and 29 are OOD data.

The mean absolute error (MAE) in HU, mean error (ME) in HU, and peak-signal-to-noise-ratio (PSNR) were computed between the sCT and ground truth CT in the patient’s external body region excluding any fixation devices. The efficiency of total uncertainty maps ($\sigma_{tot}$) (Fig. 4.D) in identifying potential error areas was assessed by comparing them to the observed absolute error maps (|CT−sCT|) (Fig. 4.C). Pearson correlation coefficients (PCC) were calculated to measure linear correlations (Fig. 3) between the uncertainty and absolute error maps. To assess the sensitivity of the PCC metric, the CT was shifted by 2 voxels translation along each axis, and the PCC was then computed to evaluate the impact of these translations.

To assess the robustness of the uncertainty estimation method, three OOD patients from three different unseen centers from the SynthRAD2023 challenge [21] were included exclusively in the testing phase. These OOD patients were not used in either the training or validation steps, ensuring that the evaluation of the uncertainty maps was based solely on data from unseen sources. Among the 26 test patients, 16 were used for dose evaluation, while OOD patients were not included in the dose assessment. Patients were treated on a VersaHD linac with two VMAT arcs. Photon plans were computed on planning CT in RaySearch RayStation v.12 A using a dose grid resolution of 2 mm. Beam parameters were then applied to the sCT, and the dose was recalculated. 3D local gamma analysis between ${\mathrm{Dose}}_{\mathrm{CT}}$ and ${\mathrm{Dose}}_{\mathrm{sCT}}$ was performed using a 1%/1 mm distance-to-agreement and a lower dose threshold of 10%. sCT uncertainty was estimated voxel-wise. A set of 50 p-sCTs was generated, constituting error scenarios for computing dose uncertainty. Fifty doses were recalculated based on the clinical treatment plan using the 50 p-sCTs. Subsequently, the dose variance map $\sigma_{dose}^{2}$ was determined from these doses.

The impact of dose uncertainty was assessed on 6 patients within our test cohort. Estimating dose uncertainty on a TPS was an exceedingly time-consuming task, so only 6 patients with distinct characteristics and a range of PCC values were selected for this analysis. These specific cases included the presence of a tracheostomy tube, prominent dental artifacts, a large tumor, and patients with irregular skin contours. The dose variance map was used to define the lower and upper bounds of the “plausible” dose range, $\left[{\mathrm{Dose}}_{\mathrm{sCT}}-3\sigma_{dose},{\mathrm{Dose}}_{\mathrm{sCT}}+3\sigma_{dose}\right]$. The interval was set to ±3σ, covering 99.7% of the data under the assumption of a normal distribution. This approach was designed to offer a reliable estimate of the possible dose variation by accounting for the majority of the uncertainty. These two dose volume histogram (DVH) curves were then overlaid with the ${\mathrm{Dose}}_{\mathrm{sCT}}$ DVH, facilitating visual comparison with the CT dose.

## 3 Results

The MAE, ME, and PSNR between sCT and reference CT within the external body were 56.5±8.2 HU, 11.1±12.9 HU, and 26.5±1.3 dB, respectively.

Fig. 3 shows the uncertainty results for different scenarios. The PCC for test patients before and after calibration (Subplot A). Calibration improves the correlation between the real error (absolute error map between sCT and ground truth CT) and the total uncertainty map (0.67 to 0.69 after calibration).

The PCC for three patients from three OOD centers are presented in Subplot A (Fig. 3). The predictions for these unseen centers show a linear correlation but are lower than those observed for the training center.

The magnitude of uncertainty for a test patient is shown in Subplot B. A significant difference was observed between patients from the training center and those from the SynthRAD database, indicating that generation quality is specific to the training center.

Fisher’s Z transformation was conducted to compare the correlation coefficients between the calibrated and uncalibrated heatmaps, focusing exclusively on patients where the correlation improved after calibration. Statistical significance was assessed using a two-tailed p-value at a significance level of α=0.0005, ensuring a strict threshold to minimize the risk of false positives in our statistical evaluation. The results indicated a significant improvement in correlation for the calibrated heatmaps, except for patient 13, where the p-value was 0.2732.

Supplementary Fig S1 illustrates the correlation between uncertainty values and absolute error values for both in-distribution and OOD patients, highlighting the differences in data distribution despite similar correlation coefficients. Fig. 4 shows the absolute error map between sCT and CT, and the total uncertainty map for five different patients. Two patients (Patients 9 and 13) were selected from the training center, while three OOD patients were chosen from another center. The sCT of Patient 28 exhibited increased noise, attributed to the CBCT image quality, which led to higher uncertainty in the sCT.

Table S1 in the supplementary material presents the dose results. The gamma pass rate (GPR) was above 98%. The standard deviation between patients’ GPR was 2.36%, indicating consistency in results across the patient cohort.

**Fig. 5:**
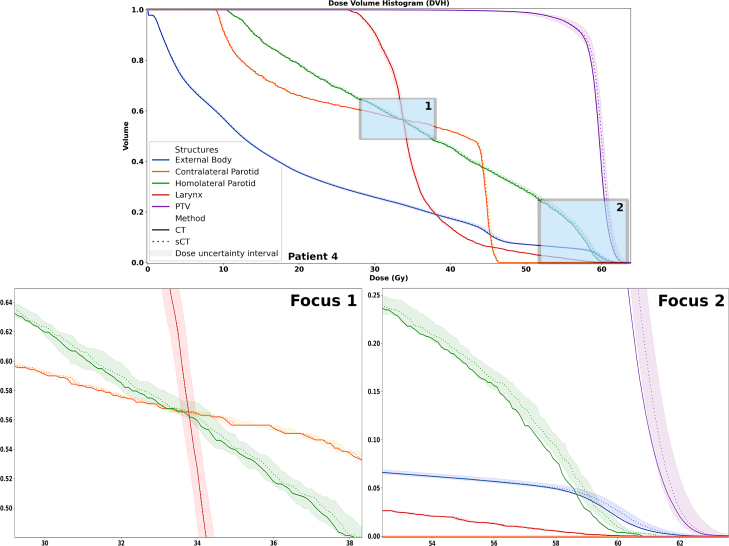
Comparison of DVH with a confidence interval (representing uncertainty) across five regions of interest for patient 8. The CT DVH is represented by a solid line, the sCT by a dashed line, and the dose uncertainty interval (confidence interval between the upper and lower bounds) by colored intervals. Patient 8’s PTV is prescribed 60 Gy delivered in fractions of 2 Gy each.

Patient DVHs were calculated in five distinct regions of interest, representing a diverse range of anatomical conditions. The DVH for the dose on the CT fell within the sCT DVH uncertainty band for all patients, except Patient 4 in high-dose regions (Fig. 5). Dose uncertainty was higher in high-dose areas with steep gradients and decreased in regions with stable dose distributions as dose levels increased.

The MAE was computed between the variance of the dose over 50 p-sCTs and the variance for each set of n sCTs, where n is 5, 10, or 25 (randomly picked from the 50 p-sCTs). Patient 8 exhibited MAE of 1.9 cGy, 1.2 cGy, and 0.6 cGy for n values of 5, 10, and 25, respectively. Patient 2’s MAE were measured at 1.8 cGy, 1.1 cGy, and 0.5 cGy for the same n values, while Patient 3 demonstrated MAE of 2.1 cGy, 1.4 cGy, and 0.6 cGy for the corresponding n values. Across all six patients, the results consistently indicated that increasing the value of n reduced the MAE between the dose variance over 50 p-sCTs and the dose variance for subsets of n p-sCTs.

## 4 Discussion

The uncertainty prediction of sCT generation from CBCT was evaluated using conventional methods and a novel approach to assess its impact on dose uncertainty. The prediction quality, measured by PCC, was tested across three unseen centers. Both model and data uncertainties were considered in estimating the impact of CT numbers uncertainty on dose. The study examines how switching from in-distribution to OOD data affects the accuracy of sCT generation.

Despite varying CBCT devices, the model performed robustly with OOD patients (PCC ∈[0.6,0.68]), slightly lower than the observed PCC during training (PCC ∈[0.65,0.72]). These results are consistent with previous studies: Van Harten et al. [12] achieved an average PCC of 0.64 for OOD patients. Law et al. [10] reported a PCC of 0.45 for the correlation between uncertainty distribution and absolute prediction error in the foreground, while Li et al. [17] attained a PCC of 0.62, and Hemsley et al. [14] reported a PCC of 0.81. In contrast, Maspero et al. [11], Rusanov et al. [13], and Karthik et al. [15] evaluated uncertainty maps only qualitatively, without quantitative metrics.

The higher uncertainty observed in OOD data [12], closely linked to CBCT quality, highlights the efficacy of our estimator in identifying OOD CBCT. This uncertainty could guide the use of alternative models, particularly for handling artifacts or poor-quality images.

Our study underlines the importance of calibrating uncertainty predictions to improve the correlation between the real error map and the uncertainty prediction map, with the PCC improving from 0.67 to 0.69 after calibration. These findings demonstrate the reliability of dose calculation from sCT for clinical application, with a GPR exceeding 98% across all patients, aligning with performance criteria from recent H&N CBCT-to-CT synthesis methods [28], [29], [30], [31].

Previous studies have also explored the use of sCT uncertainty for clinical decision-making in proton therapy. Galapon et al. [32] demonstrated a correlation between uncertainty maps and dose difference maps between CT and sCT, focusing specifically on the area covered by the 5% isodose curve. Li et al. [17] incorporated sCT uncertainty predictions into robust proton therapy optimization by using three different sCTs and introduced an uncertainty-aware DVH analysis. Our approach proposes a method for estimating dose uncertainty directly from sCT uncertainties, thereby validating sCTs for clinical use while accounting for potential image inaccuracies and their implications for treatment planning.

The dose uncertainty estimate is relatively low due to a MAE of 56.5±8.2 HU for sCTs, comparable to the top methods in the SynthRAD2023 challenge [21] in the H&N region. Additionally, photon-based treatment further reduces dose uncertainty because photon dose calculations are less sensitive to sCT inaccuracies compared to proton therapy. However, significant dose uncertainty exists in high-dose regions due to the steep dose gradients in these areas. Despite inaccuracies in the DVH estimation for Patient 4’s high-dose areas, the uncertainty interval effectively predicted dose variance due to sCT numbers errors in all other cases (Supplementary Fig S2–S3). Reducing the number of sCT samples decreased the dose variance difference between 25 and 50 samples to sub-cGy levels, thereby improving time efficiency.

For practical use, uncertainty maps provide valuable insights to aid decision-making in ART and dose accumulation. These maps visually and quantitatively highlight regions where sCT predictions may be less reliable, providing clinicians with valuable information to assess the potential impact on treatment delivery, enabling adjustments to the treatment plan or verification process if needed, in response to anatomical changes or imaging inconsistencies. In dose accumulation scenarios, uncertainty maps can be used to take into account the cumulative uncertainties over several fractions when patient anatomy and CBCT quality change over time. By establishing a confidence interval around calculated doses, these maps quantify deviations from the planned dose, adding a layer of safety. Clinicians can leverage these intervals, rather than relying on a single sCT, to more accurately assess dose calculations.

This study has limitations, including time complexity in determining dose uncertainty and the lack of a comprehensive OOD image analysis (from unseen center). While our estimator has been empirically evaluated, studying its properties would be interesting, though complicated by the image size and the nonlinear nature of the network.

Our probabilistic methods effectively predict sCT uncertainty, which correlates with the actual error (CT/sCT). Additionally, these results suggest that total uncertainty maps are effective for assessing sCT quality. This work presents a novel approach for obtaining a DVH confidence interval around the CT DVH using a MC method to estimate the impact of generation uncertainties on the dose.

## 5 Compliance with ethical standards

For this study, 85 H&N patients were retrospectively selected from the ARTIX study (Adaptive Radiotherapy to Decrease Xerostomia in Oropharynx Carcinoma), which was approved by the French Institutional Review Board and registered on ClinicalTrials.gov (NCT01874587) as of July 2013.

For the SynthRAD dataset, each institution received ethical approval from their internal review board/Medical Ethical committee as follows: UMC Utrecht classified the study as non-WMO on March 4, 2022, under approval number 22/474. UMC Groningen classified the study as non-WMO on July 20, 2022, under approval number 202200310. Radboudumc declared the study non-WMO on October 17, 2022, under approval number 2022–15950.

## CRediT authorship contribution statement

**Cédric Hémon:** Conceptualization, Methodology, Writing – original draft. **Lucía Cubero:** Methodology, Data curation, Writing – review & editing. **Valentin Boussot:** Conceptualization, Methodology, Writing – review & editing. **Romane-Alize Martin:** Methodology, Writing – review & editing. **Blanche Texier:** Methodology, Writing – review & editing. **Joël Castelli:** Writing – review & editing, Supervision. **Renaud de Crevoisier:** Resources, Writing – review & editing, Supervision, Funding acquisition. **Anaïs Barateau:** Conceptualization, Writing – review & editing, Supervision, Project administration. **Caroline Lafond:** Conceptualization, Writing – review & editing, Supervision, Project administration, Funding acquisition. **Jean-Claude Nunes:** Conceptualization, Resources, Writing – review & editing, Supervision, Project administration, Funding acquisition.

## Declaration of competing interest

The authors declare the following financial interests/personal relationships which may be considered as potential competing interests: Cedric Hemon reports financial support was provided by Elekta AB. If there are other authors, they declare that they have no known competing financial interests or personal relationships that could have appeared to influence the work reported in this paper.

## Appendix A Supplementary data

MMC S1 Supplementary material provides insights into sCT generation methods, error-uncertainty correlation, dose evaluation metrics, and DVH uncertainty analysis.
