# Supplementary material for: Modeling dose uncertainty in cone-beam computed tomography: Predictive approach for deep learning-based synthetic computed tomography generation
Source: Phys Imaging Radiat Oncol. 2025 Jan 26;33:100704. doi: 10.1016/j.phro.2025.100704 (PMC11815694; doi:10.1016/j.phro.2025.100704)
Supplement: MMC S1 — Supplementary material provides insights into sCT generation methods, error-uncertainty correlation, dose evaluation metrics, and DVH uncertainty analysis. [file mmc1.pdf]

1. Supplementary Material

1.1. CBCT-to-CT synthesis

The second output channel of the network predicts the standard deviation of the input's noise, representing the uncertainty map associated with the data (σ_{data}). The generator was a six-block ResNet, whereas a PatchGAN [1] was used as the discriminator. The discriminator's loss function was based on binary cross-entropy. A style transfer approach was used to address the sCT generation problem. The sCT was reconstructed with the content of the CBCT by applying the style of the CT using a perceptual loss (PL) [2]. An additional uncertainty loss $\mathcal{L}_{unc}$, detailed in Section 2.4, was introduced to predict the uncertainty map and apply a constraint on the sCT prediction. The PL loss employed in this study was the CREPs loss (Content and Style Representation by an Enhanced Perceptual synthesis) [3, 4] proposed in the SynthRAD2023 MICCAI Challenge. The loss of style and content involves the projection of the input and output images into a feature space specifically designed to encapsulate either texture details or shape information, such as anatomy. This feature space relies on a neural network pre-trained for object recognition using the ImageNet dataset [5].

1.2. Results

Patient		2	3	4	8	9	10	11	12
GPR(%)		99.8	99	96	99.8	99.3	98.2	98.7	98.6
Mean Gamma		0.06	0.13	0.18	0.11	0.10	0.16	0.14	0.14
$MAE_{dose}(cGy)$		1.5	4.0	13.0	4.2	4.0	5.9	4.4	4.6
Patient		13	18	20	21	22	23	24	25
GPR(%)		98.3	99.6	99.5	99.4	90	99.5	99.7	99.3
Mean Gamma		0.15	0.08	0.08	0.11	0.13	0.09	0.09	0.08
$MAE_{dose}(cGy)$		5.1	2.4	2.5	2.9	3.3	4.7	4.5	2.1

Table S1. Gamma pass rate (GPR) (3D local gamma analysis, 1%/1 mm distance to agreement and a dose threshold of 10%), mean gamma values, and MAE_{dose} (in cGy) were calculated to compare the total dose distribution on the reference CT with that on the sCTs. Patients 8 and 9 received a total dose of 60 Gy, delivered in 2 Gy per fraction. Similarly, patients 2, 3, 21, and 22 were also prescribed 60 Gy with 2 Gy per fraction. Patient 4 had a slightly higher prescription of 69.9 Gy, delivered in 2.12 Gy per fraction. Patients 12, 13, 20, 23, and 24 were prescribed 63 Gy with 1.8 Gy per fraction. Patients 10 and 11 received 72.4 Gy with 2.07 Gy per fraction. Patient 18 was prescribed 64.4 Gy with 1.84 Gy per fraction, while patient 25 received 55 Gy with 1.83 Gy per fraction.

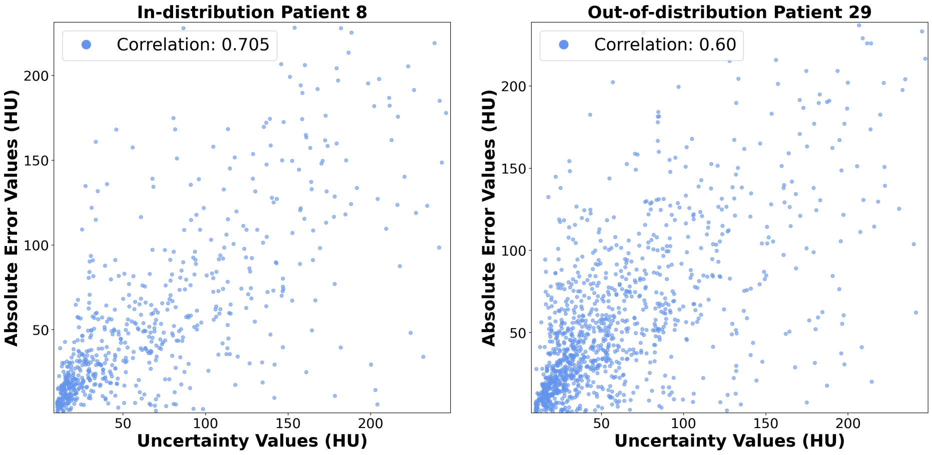

Figure S1. Scatter plots between uncertainty and absolute error for In-Distribution and OOD patients. These scatter plots display the relationship between uncertainty values (HU) and corresponding absolute errors (HU) for in-distribution (left, Patient 8) and out-of-distribution (right, Patient 29) cases. Each point represents a measurement of uncertainty plotted against the associated absolute error.

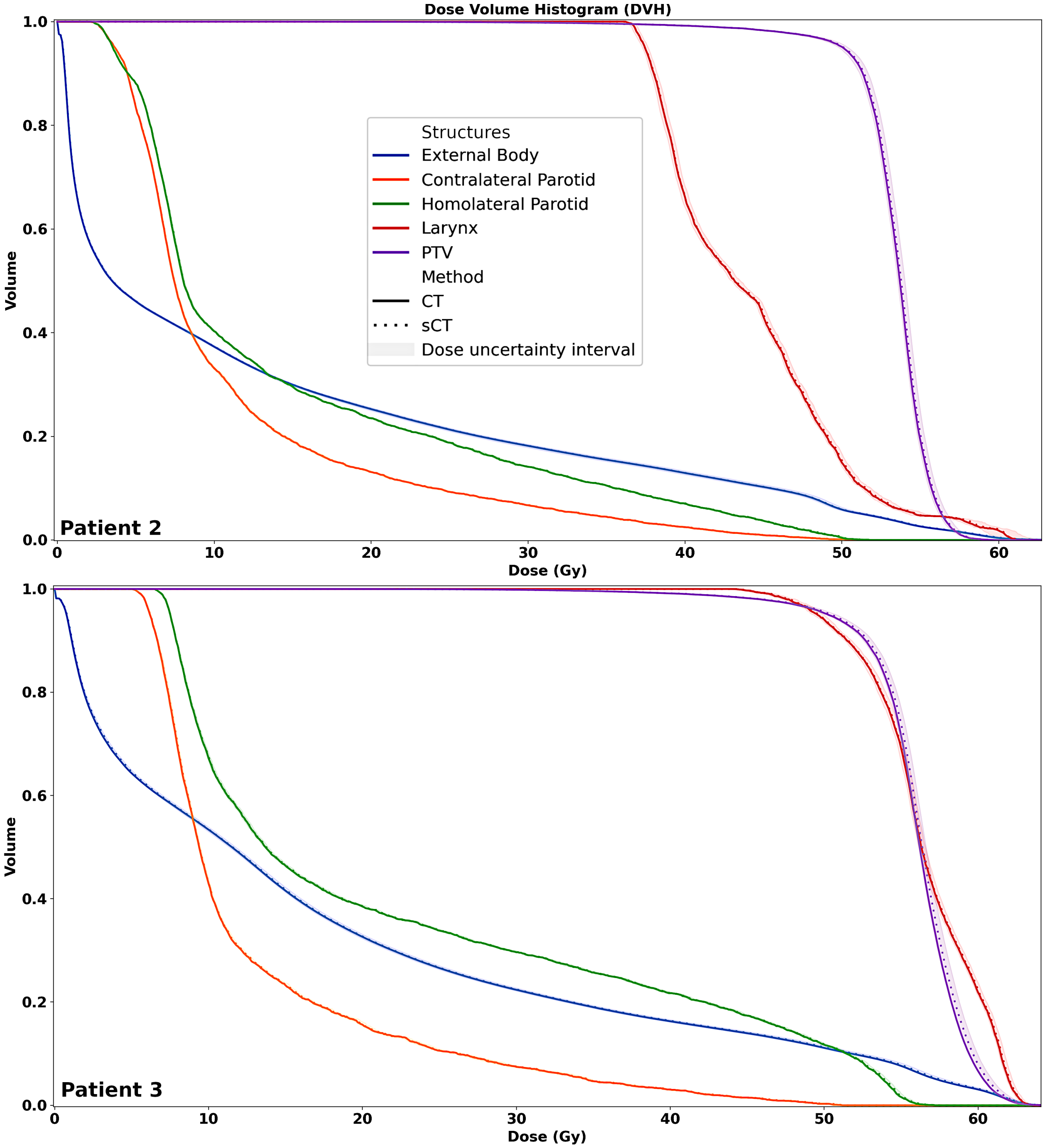

Figure S2. Comparison of DVH with a confidence interval (representing uncertainty lower and upper bound) across five regions of interest in two different patients (2 and 3). The CT DVH is represented by a solid line, the sCT by a dashed line, and the dose uncertainty interval (confidence interval between the upper and lower bounds) by colored intervals. Patients 2's and 3's PTV are prescribed 60 Gy, delivered in fractions of 2 Gy each.

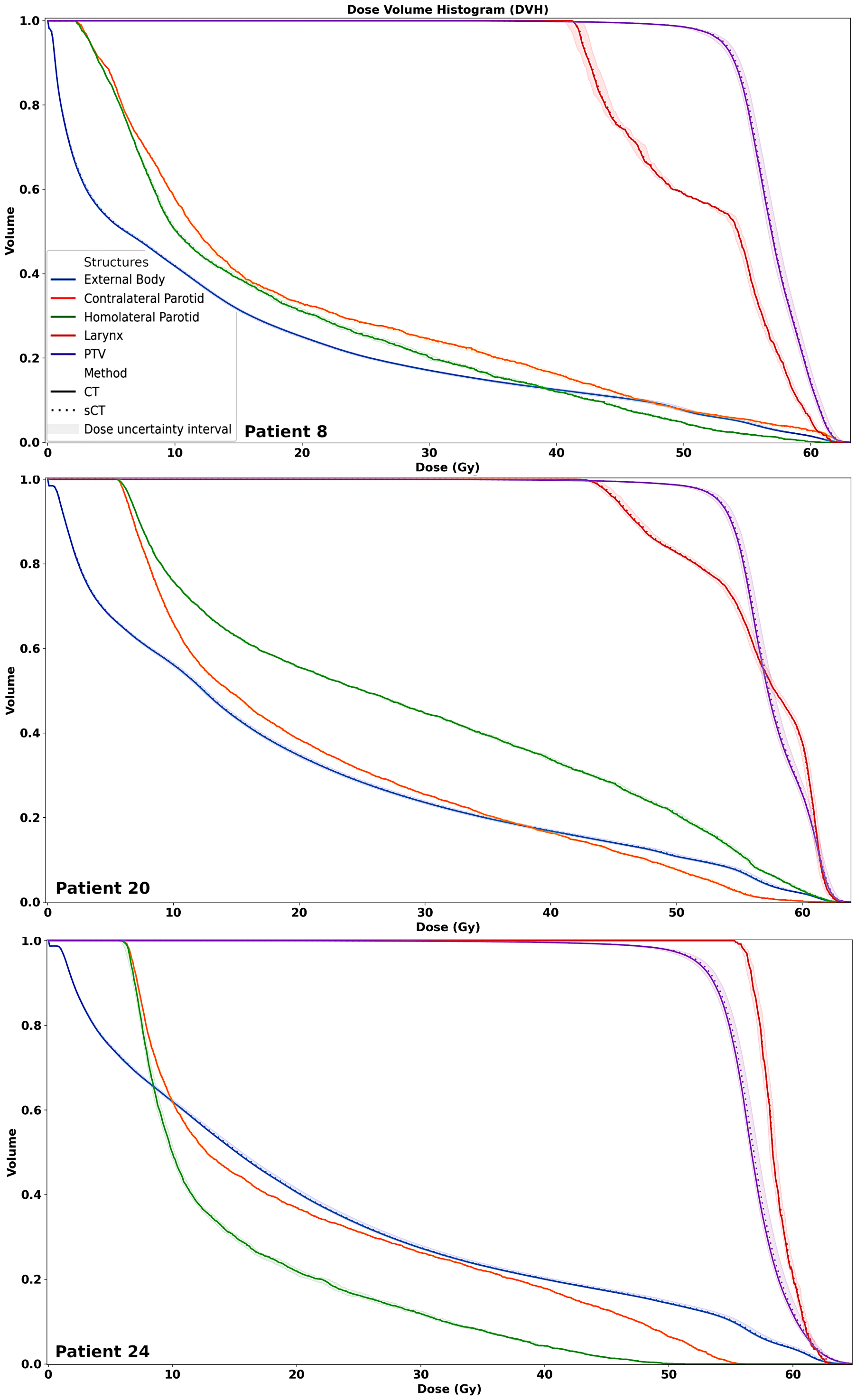

Figure S3. Comparison of DVH with a confidence interval (representing uncertainty lower and upper bound) across five regions of interest in two different patients (8, 20 and 24). The CT DVH is represented by a solid line, the sCT by a dashed line, and the dose uncertainty interval (confidence interval between the upper and lower bounds) by colored intervals. Patient 8's PTV is prescribed 60 Gy, delivered in fractions of 2 Gy each. Patients 20 and 24 are prescribed 63 Gy, delivered in fractions of 1.8 Gy each.

References

[1] Isola P, Zhu JY, Zhou T, Efros AA. Image-to-Image Translation with Conditional Adversarial Networks in: PROC CVPR IEEE 2018 p. 1125–1134. doi:10.48550/arXiv.1611.07004.

[2] Johnson J, Alahi A, Fei-Fei L. Perceptual Losses for Real-Time Style Transfer and Super-Resolution. Lect Notes Comput SC 2016; 9906: 694–711. doi:10.1007/978-3-319-46475-6_43.

[3] Hémon C, Boussot V, Texier B, Dillenseger JL, Nunes JC Guiding Unsupervised CBCT-to-CT synthesis using Content and style Representation by an Enhanced Perceptual synthesis (CREPs) loss in: SynthRAD2023 Challenge, MICCAI 2023, Vancouver (BC), Canada 2023. doi:hal-04272509.

[4] Huijben E, Terpstra ML, Galapon Jr A, Pai S, Thummerer A, Koopmans P et al. Preprint generating Synthetic Computed Tomography for Radiotherapy: SynthRAD2023 Challenge Report. Med Image Anal 2024;: . doi:10.1016/j.media.2024.103276.

[5] Deng J, Dong W, Socher R, Li LJ, Li K, Fei-Fei L ImageNet: A large-scale hierarchical image database in: PROC CVPR IEEE 2009 p. 248–255. doi:10.1109/CVPR.2009.5206848.
